# NEUROPSI battery subtest profile in subcortical vascular dementia and Alzheimer's disease

**DOI:** 10.1590/S1980-57642012DN06030010

**Published:** 2012

**Authors:** Maria Niures P.S. Matioli, Paulo Caramelli

**Affiliations:** 1 Department of Geriatrics, Lusíada University School of Medicine, Santos SP, Brazil; 2 Post-Graduate Program, Department of Neurology, University of São Paulo School of Medicine, São Paulo SP, Brazil; 3 Behavioral and Cognitive Neurology Research Group, Department of Internal Medicine, Faculty of Medicine of the Federal University of Minas Gerais, Belo Horizonte MG, Brazil

**Keywords:** NEUROPSI, vascular dementia, Alzheimer's disease, memory recall, verbal fluency, neuropsychological tests

## Abstract

**Objective:**

To investigate the diagnostic value of subtests of the NEUROPSI battery for differentiating subcortical vascular dementia (SVaD) from Alzheimer's disease (AD).

**Methods:**

Thirteen patients with mild SVaD, 15 patients with mild probable AD, and 30 healthy controls, matched for age, education and dementia severity (in the case of patients), were submitted to the Mini-Mental State Examination (MMSE) and NEUROPSI battery. The performance of AD and SVaD groups on NEUROPSI subtests was compared. The statistical analyses were performed using Kruskal-Wallis, Chi-square and Mann-Whitney tests. The results were interpreted at the 5% significance level (p<0.05). Bonferroni's correction was applied to multiple comparisons (α=0.02).

**Results:**

SVaD and AD patients showed no statistical difference in MMSE scores (SVaD=20.8 and AD=21.0; p=1.0) or in NEUROPSI total score (SVaD=65.0 and AD=64.3; p=0.56), suggesting a similar severity of dementia. The AD group performed worse on memory recall (<0.01) and SVaD group was worse in verbal fluency subtests (p=0.02).

**Conclusion:**

NEUROPSI's memory and language subtests can be an auxiliary tool for differentiating SVaD from AD.

## Abstract (pt)

**Objetivo:**

Investigar o valor diagnóstico dos substes da bateria NEUROPSI em diferenciar demência vascular subcortical (DVs) de doença de Alzheimer (DA).

**Métodos:**

Treze pacientes com demência vascular subcortical leve, 15 pacientes com diagnóstico de DA provável leve e 30 indivíduos controles saudáveis, pareados em relação à idade, escolaridade e gravidade da demência, foram submetidos ao Mini-Exame de Estado Mental (MEEM) e a bateria NEUROPSI. O desempenho dos grupos DA e DVs nos subtestes do NEUROPSI foi comparado. As análises estatísticas foram feitas através dos testes de Kruskal-Wallis, qui-quadrado e Mann-Whitney, os reultados foram interpretados com 5% do nível de significância (p<0,05). A correção de Bonferroni foi aplicada para múltiplas comparações (α=0,02).

**Resultados:**

Pacientes dos grupos DVs e DA não mostraram diferenças estatísticas no desempenho do MEEM (DVs=20,8 e DA=21,0; p=1,0) e na pontuação total do NEUROPSI (DVs=65,0 e DA=64,3; p=0,56), sugerindo similar gravidade da demência. O grupo DA apresentou pior desempenho em evocação da memória (<0,01) e o grupo DVs foi pior nos subtestes de fluência verbal (p=0,02).

**Conclusão:**

Os subtestes de memória e de linguagem do NEUROPSI podem ser uma ferramenta auxiliar na diferenciação entre DVs e DA.

## INTRODUCTION

In a report issued in 2012, the World Health Organization estimates there were 35.6 million people living with dementia worldwide in 2010 and that this number will increase to 65.7 million by 2030, with two-thirds of cases living in low and middle-income countries, including Brazil and other Latin American countries.^1^ In Latin America, Nitrini et al. showed the global prevalence of dementia was 7.1% in individuals aged 65 years or older, mirroring the rates of developed countries, but with higher prevalence in relatively younger subjects (65-69 years).^2^

Alzheimer disease (AD) and vascular dementia (VaD) are the most common causes of dementia in the elderly.^2^ The most common subtype of VaD is subcortical ischemic vascular dementia (SVaD), which has slow onset and a gradual clinical course, with or without acute motor or sensory deficits.^3^ SVaD is characterized by executive dysfunction and mild memory impairment.^4,5^ In the early stages, AD shows impairment of declarative memory, particularly in consolidation of information into long-term memory, resulting in accelerated forgetting and poor delayed recall.^6^ Deficits in immediate and episodic memory as well as in language (e.g., naming) are common in AD.^6^

Neuropsychological evaluation is an important tool for characterizing cognitive deficits, especially to differentiate normal cognition from mild cognitive impairment and dementia. Cognitive evaluation is widely recommended by the literature in international and national consensus for dementia diagnosis, particularly in AD^7,8^ and VaD.^3,5^

The NEUROPSI is a brief neuropsychological battery developed to assess a wide spectrum of cognitive functions, namely, orientation, attention and concentration, memory, language, reading and writing, visuoperceptual abilities and executive functions.^9^ This battery was developed especially for use in Latin American people to enable neuropsychological evaluation adapted to the social and cultural characteristics of this population.^9^ The NEUROPSI battery was standardized for Spanish speaking Latin America in Mexico^9^ and translated into Portuguese for use in Brazil by Abrisqueta-Gómez et al.^10^

In the present study, we compared the performance of SVaD and AD patients on NEUROPSI's subtests aiming to determine whether they can be useful for differential diagnosis in a clinical setting.

## POPULATION AND METHODS

We studied 58 individuals, aged 50 years or older, who were patients and healthy volunteers from two teaching hospitals, the Geriatric Outpatient Clinic of Guilherme álvaro Hospital in Santos and the Cognitive Neurology Outpatient Clinic from the Hospital das Clínicas of the University of São Paulo School of Medicine in São Paulo, Brazil.

The sample was divided into three groups: controls without cognitive impairment and free from neurological and psychiatric diseases; patients with SVaD according to DSM-IV^11^ diagnostic criteria for VaD with the presence of vascular leukoencephalopathy or Binswanger's disease and/or, subcortical multiple lacunes on brain magnetic resonance imaging (MRI);^12^ and probable AD patients according to NINCDS-ADRDA criteria.^13^ All SVaD and AD patients were submitted to appropriate laboratory tests and to MRI.^14^

To rule out depression, the controls were submitted to the Geriatric Depression Scale (GDS)^15^ and patients to the Cornell scale (cut-off ≥7) for depression in dementia.^16^ The Jeste and Finkel criteria for psychosis of AD and related dementias^17^ were applied to SVaD and AD groups to exclude psychosis.

The three groups were matched for age, gender and education. The Mini-Mental State Examination (MMSE) with education-adjusted scores^18^ and Pfeffer Functional Activities Questionnaire (PFAQ)^19^ were administered to all participants as part of the diagnostic workup. SVaD and AD patients had mild dementia, according to MMSE scores. The subjects were assigned to the control group when they had PFAQ <5,^19^ GDS<10,^15^ and MMSE cut-off score according to years of schooling: illiterate ≥20, 1-4 years ≥25, 5-8 years ≥26, 9-11 years ≥28 and >11 years ≥29.^18^

The Hachinski Ischemic Scale (HIS)^20^ was only administered to the demented groups. The NEUROPSI was applied to all subjects and the total score of each group was determined. However, scores for NEUROPSI's subtests were only calculated for the SVaD and AD groups.

**Characteristics of NEUROPSI subtests^10^.** Orientation: evaluating the subject's temporal and spatial orientation (date and place) and personal data (age).

*Attention and concentration:* Tested by reverse repetition of digits and mental control tasks, such as serially subtracting 3 (starting at 20, five consecutive times); cancellation tasks are used to examine spatial hemineglect or visual negligence processes.

*Memory:* Information encoding, storage, and retrieval processes are tested. Verbal memory tasks consist of a series of 6 words read aloud (two animals, two fruits, and two body parts), which subjects are asked to repeat; three trials are included after 20 minutes (without previous warning) the six words are recalled (free recall), then a number of clues are provided to increase recall according to the semantic content of the word (cued recall), and finally word recognition is required. For the visual test, the subject is asked to copy a drawing of a semicomplex figure (Rey's figure adapted for the battery); without previous warning, the drawing is recalled after 20 minutes.

*Language:* Includes semantic fluency (animals/minute) and phonologic fluency (letter F/minute) tests. The comprehension subtest comprises a sheet with 4 figures (two circles and two squares, large and small), on which the subject is asked to mark the figure following verbal instructions. Repetition subtest requires subjects to repeat certain words or sentences. In the Naming subtest, subjects are shown eight figures (one at a time) of several objects and asked to name them. Reading and writing: reading a story aloud and then answering three questions about it. Dictation: the subject has to write the sentence which is read aloud. Copy: the subject is asked to copy a sentence supplied in the test.

*Executive functions:* Divided into conceptual and motor subtests. Conceptual tasks include identifying similarities between pairs of stimuli (animals, fruits, parts of the body). Other tasks in this subtest include solving some mental arithmetic operations and continuing a sequence of circles and crosses. Executive motor functions test: the subject is asked to reproduce three consecutive movements changing the position of the hands (initially with the right hand and then with the left), then to perform alternate movements with both hands and finally to respond to opposite stimuli (for instance, when a finger is shown, the subjects must respond with a fist and vice versa).

**Statistical analysis.** Data were analyzed with SPSS (Statistical Package for Social Sciences version 14.0) software. The three groups were compared on socio-demographic variables and neuropsychological scores by the Kruskal-Wallis test, due to the non-normal distribution of these variables. The Chi-square test was applied to gender whereas the Mann-Whitney test was employed to compare scores of SVaD and AD groups. All statistical tests were interpreted at the 5% significance level (p<0.05). Bonferroni's correction was applied to multiple comparisons (α=0.02) among AD and SVaD groups because of the small number of subjects tested, and p values ≤0.02 were then considered significant.

## RESULTS

Thirteen SVaD patients (four female and nine male; mean age=68.0 years; mean schooling=7.2 years), 15 AD patients (10 female and five male; mean age=76.0 years; mean schooling=5.8 years) and 30 controls (19 female and 11 male; mean age=72.3 years; mean schooling=7.0 years) were evaluated. The three groups were adequately matched for age, gender and years of education (Table 1). The SVaD group had no hippocampal atrophy on MRI. Four out of the 15 AD patients had very slight subcortical white-matter changes in the periventricular regions on MRI, and all subjects in the AD group had hippocampal atrophy.

**Table 1:** Gender, age, education, MMSE and Pfeffer's Functional Activities Questionnaire scores in the three diagnostic groups

	Controls	SVaD	AD	P value
Gender	F=19M=11	F=4 M=9	F=10 M=5	0.09
Age	72.3 (sd=7.5)	68 (sd=12)	76 (sd=7.1)	0.21
Education	7 (sd=4.2)	7.2 (sd=4.9)	5.8 (sd=3.4)	0.57
MMSE	28.5 (sd=1.6)	20.8 (sd=3.2)	21 (sd=3.3)	<0.01*1.0^#^
Pfeffer questionnaire	0.1 (sd=0.4)	17.9 (sd=5.6)	12.8 (sd=5.3)	<0.01*0.02^#^
HIS		8.2 (sd=2.5)	1.9 (sd=1.1)	0.01
NEUROPSI	102.3 (sd=13.2)	64.2 (sd=8.6)	63.6 (sd=12.1)	<0.01*0.56^#^

F: female; M: male; MMSE: Mini-Mental State Examination; AD: Alzheimer's disease; SVaD: Subcortical vascular dementia; sd: standard deviation;

* patients (SVaD and AD) vs. controls;

# SVaD vs. AD.

The scores on the MMSE (p<0.01), PFAQ (p<0.01) and NEUROPSI (p<0.01) differed among the three groups (Table 1). Moreover, SVaD and AD patients showed no statistical difference in MMSE or in NEUROPSI total score, suggesting similar severity of dementia. Scores on the PFAQ (p=0.02) and HIS (p<0.01) were higher in the SVaD than in the AD group (Table 1).

Significant differences were found between the two dementia groups in total score on verbal fluency tasks (p=0.02), including the raw scores of their semantic (p<0.01) and phonemic (p<0.01) subcategories, and memory recall tasks (p<0.01) from the NEUROPSI. No additional differences between SVaD and AD patients were found on NEUROPSI subtests (Table 2).

**Table 2:** NEUROPSI Subtest scores for SVaD and AD groups.

Subtests			SVaD	AD	P value
1.	Orientation		4.1 (sd=1.3)	4.5 (sd=1.2)	0.46
2.	Attention/concentration		12.9 (sd=2.9)	13.8 (sd=3.6)	0.20
	a.	reverse repetition of digits	2.6 (sd=0.7)	2.7 (sd=1.1)	0.76
	b.	visual detection	5.8 (sd=1.4)	6.9 (sd=2.7)	0.27
	c.	calculation	3.7 (sd=1.4)	4.2 (sd=0.9)	0.42
3.	Memory		9.1 (sd=3.5)	11.1 (sd=2.9)	0.25
	a.	spontaneous memory	3.5 (sd=0.9)	3.5 (sd=0.7)	0.75
	b.	copy of a figure	5.7 (sd=3.5)	7.7 (sd=2.7)	0.19
4.	Language		18.2 (sd=1.5)	18.9 (sd=2.2)	0.33
	a.	naming	7.5 (sd=0.5)	7.2 (sd=0.8)	0.45
	b.	repetition	4 (sd=0)	3.9 (sd=0.3)	0.76
	c.	comprehension	4.4 (sd=1)	4.6 (sd=1.4)	0.43
	d.	verbal fluency	2.3 (sd=0.6)	3.2 (sd=1.0)	0.02
		semantic fluency	6.5 (sd=2.1)	10.2 (sd=4.2)	<0.01
		phonologic fluency	3.3 (sd=2.6)	7.3 (sd=4.1)	<0.01
5.	Reading		0.8 (sd=0.7)	0.4 (sd=0.6)	0.10
6.	Writing		1.4 (sd=0.8)	1.3 (sd=0.9)	1.00
7.	Executive functions		11.2 (sd=2.6)	10.5 (sd=3.4)	0.55
	a.	conceptual			
		similarities	2.7 (sd=2.2)	1.4 (sd=1.6)	0.12
		calculation	1.7 (sd=0.9)	1.8 (sd=0.90)	0.85
		sequence	0.1 (sd=0.3)	0.2 (sd=0.4)	0.58
	b.	motor functions			
		changing the position of hands	3.7 (sd=1.1)	3.6 (sd=1.1)	0.82
		alternative movements of hands	1.3 (sd=0.9)	1.7 (sd=0.5)	0.45
		opposite stimuli response	1.8 (sd=0.6)	1.8 (sd=0.4)	0.89
8.	Memory recall (after 20 minutes)		8.2 (sd=2.9)	4.3 (sd=2.8)	<0.01
	a.	visual memory			
		(recall the copy of figure)	2.4 (sd=2.4)	0.4 (sd=0.7)	0.02
	b.	verbal memory			
		free recall	0.5 (sd=0.8)	0.1 (sd=0.3)	0.14
		cued recall	1.0 (sd=0.9)	0.7 (sd=0.9)	0.29
		word recognition	4.2 (sd=1.9)	3.2 (sd=1.9)	0.19
9.	Total score		65.0 (sd=8.6)	64.3 (sd=11.7)	0.56

AD: Alzheimer's disease; SVaD: Subcortical vascular dementia; sd: standard deviation.

## DISCUSSION

The NEUROPSI battery was described by Ostrosky-Solis et al.^9^ as an efficient test for detecting cognitive impairment in AD patients and for differentiating between initial and intermediate stages of this type of dementia. Subsequently, Abrisqueta-Gomez et al.^10^ showed that the Portuguese version of the NEUROPSI had the same ability to identify normal cognition from initial and intermediate stages of AD.

In our study, the test proved able to discriminate subjects with dementia (AD and SVaD) from cognitively healthy controls as shown previously by Abrisqueta-Gomez et al.^10^ Total score on the NEUROPSI was unable to differentiate the performance of AD and SVaD groups. In the present work, NEUROPSI subtests were analyzed independently and memory recall and verbal fluency tasks showed different performance between SVaD and AD patients. SVaD subjects had worse performance on verbal fluency tasks, while AD patients were worse on memory recall. These results can be explained by the hallmarks of AD dementia, i.e., memory and language impairment due to pathological lesions first involving the entorhinal cortex and the hippocampus, temporal limbic structures and reciprocal corticolimbic connections.^6,21^ SVaD is mainly characterized by impairment in executive functions with memory being only slightly affected.^3,5,22^ Phonemic verbal fluency is a good test for assessing executive functions and the integrity of prefrontal cortex. Bilateral prefrontal and dorsolateral cortices and ventral median areas, and inferior-lateral temporal lobe have been described as important areas involved in the semantic verbal fluency process.^23-25^ SVaD predominantly affects the prefrontal subcortical circuit, which explains the occurrence of the cognitive profiles seen in our SVaD group.^4,26^

We failed to find significant differences between SVaD and AD groups on other executive tasks of the NEUROPSI battery, probably because executive dysfunction also occurs in dementia due to AD.^8,21^

There are numerous limitations in the ability of cognitive tests to discriminate between AD and VaD. Hence, cognitive tests must be used cautiously and only in conjunction with other information (medical history, neuroimaging and, ultimately and more recently, with biomarkers) to define the specific cause of cognitive impairment or dementia.^5,8,27^ This research area is further limited by the coexistence of mixed pathology (AD and VaD) in a substantial proportion of patients, especially in older populations.^3^

In conclusion, NEUROPSI's memory and language subtests can be an auxiliary tool for differentiating SVaD from AD. However, the small sample sizes of the present study preclude the generalization of our findings and replication of results by additional studies is necessary.

**Declaration.** The authors have no conflicts of interest to declare regarding this research, which was carried out without financial support.

## References

[1] World Health Organization (2012). Dementia: a public health priority.

[2] Nitrini R, Bottino CM, Albala C (2009). Prevalence of dementia in Latin America: a collaborative study of
population-based cohorts. Int Psychogeriatr.

[3] Gorelick PF, Scuteri A, Black SE (2011). Vascular contributions to cognitive impairment and dementia: a
statement for healthcare professionals from the American Heart
Association/American Stroke Association. Stroke.

[4] Yuspeh RL, Vanderploeg RD, Crowell TA, Mullan M (2002). Differences in executive functioning between Alzheimer's disease
and subcortical ischemic vacular dementia. J Clin Exp Neuropsychol.

[5] Engelhardt E, Tocquer C, André C, Moreira DM, Okamoto IH, Cavalcanti JLS (2011). Demência vascular: Critérios diagnósticos e
exames complementares. Dement Neuropsychol.

[6] Lindeboom J, Weinstein H (2004). Neuropsychology of cognitive ageing, minimal cognitive
impairment, Alzheimer's disease, and vascular cognitive
impairment. Eur J Pharmacol.

[7] McKhann GM, Knopman DS, Chertkow H (2011). The diagnosis of dementia due to Alzheimer's disease:
Recommendations from the National Institute on Aging-Alzheimer's Association
workgroups on diagnostic guidelines for Alzheimer's disease. Alzheimers Dement.

[8] Chaves MLS, Godinho CC, Porto CS (2011). Doença de Alzheimer: avaliação cognitiva,
comportamental e emocional. Dement Neuropsychol.

[9] Ostrosky-Solis F, Ardila A, Rosselli M (1999). NEUROPSI: a brief neuropsychological test battery in Spanish with
norms by age and educational level. J Int Neuropsychol Soc.

[10] Abrisqueta-Gomez J, Ostrosky-Sollis F, Bertolucci PH, Bueno OF (2008). Applicability of the abbreviated neuropsychologic battery
(NEUROPSI) in Alzheimer disease patients. Alzheimer Dis Assoc Disord.

[11] American Psychiatric Association (1994). Diagnostic and Statistical Manual of Mental Disorders (DSM-IV).

[12] Engelhardt E, Moreira DM, Alves G (2006). Demência vascular: os grandes subtipos
clínico-patológicos isquêmicos. Rev Bras Neurol.

[13] Mckhann G, Drachman D, Folstein M, Katzman R, Price D, Stadlan EM (1984). Clinical diagnosis of Alzheimer's disease: report of the
NINCDS-ADRDA work group under the auspices of the Department of Health &
Human Services Task Force on Alzheimer's disease. Neurology.

[14] Nitrini R, Caramelli P, Bottino CM, Damasceno BP, Brucki SMD, Anghinah R (2005). Diagnosis of Alzheimer's disease in Brazil: diagnostic criteria
and auxiliary tests. Recommendations of the Scientific Department of
Cognitive Neurology and Aging of the Brazilian Academy of
Neurology. Arq Neuropsiquiatr.

[15] Stoppe Júnior A, Jacob Filho W, Louzã Neto MR (1994). Avaliação de depressão em idosos
através da Escala de Depressão em Geriatria: resultados
preliminares. Rev ABP-APAL.

[16] Carthery-Goulart MT, Areza-Fegyveres R, Schultz RR (2007). Brazilian version of the Cornell depression scale in
dementia. Arq Neuropsiquiatr.

[17] Jeste DV, Finkel SI (2000). Psychosis of Alzheimer's disease and related dementias.
Diagnostic criteria for a distinct syndrome. Am J Geriatr Psychiatry.

[18] Brucki SMD, Nitrini R, Caramelli P, Bertolucci PHF, Okamoto IH (2003). Suggestions for utilization of the mini-mental state examination
in Brazil. Arq Neuropsiquiatr.

[19] Pfeffer RI, Kurosaki TT, Harrah CH, Chance JM, Filis S (1982). Measurement of functional activities in older adults in the
community. J Gerontol.

[20] Hachinski VC, Lanssen NA, Marshall J (1974). Multi-infarct dementia: a cause of mental deterioration in the
elderly. Lancet.

[21] Mayeux R (2010). Early Alzheimer's Disease. N Engl J Med.

[22] O'Brien JT, Erkinjuntti T, Reisberg B (2003). Vascular cognitive impairment. Lancet Neurol.

[23] Costafreda SG, Fu CHY, Lee L, Everitt B, Brammer MJ, David S (2006). A systematic review and quantitative appraisal of fMRI studies of
verbal fluency: role of the left inferior frontal gyrus. Hum Brain Mapp.

[24] Szatkowska I, Grabowska A, Szymanska O (2000). Phonological and semantic fluencies are mediated by different
regions of the prefrontal cortex. Acta Neurobiol Exp.

[25] Altmann LJP, McClung JS (2008). Effects of semantic impairment on language use in Alzheimer's
disease. Semin Speech Lang.

[26] Canning SJ, Leach L, Stuss D, Ngo L, Black SE (2004). Diagnostic utility of abbreviated fluency measures in Alzheimer
disease and vascular dementia. Neurology.

[27] Mathias JL, Burke J (2009). Cognitive functioning in Alzheimer's and vascular dementia: A
meta-analysis. Neuropsychology.

